# β Cell GHS-R Regulates Insulin Secretion and Sensitivity

**DOI:** 10.3390/ijms22083950

**Published:** 2021-04-11

**Authors:** Geetali Pradhan, Chia-Shan Wu, Daniel Villarreal, Jong Han Lee, Hye Won Han, Akhilesh Gaharwar, Yanan Tian, Wenxian Fu, Shaodong Guo, Roy G. Smith, Yuxiang Sun

**Affiliations:** 1 USDA/ARS Children’s Nutrition Research Center, Department of Pediatrics, Baylor College of Medicine, Houston, TX 77030, USA; geetali.pradhan@alumni.bcm.edu (G.P.); jhleecw@gmail.com (J.H.L.); 2 Interdepartmental Program in Translational Biology and Molecular Medicine, Baylor College of Medicine, Houston, TX 77030, USA; 3 Department of Nutrition, Texas A&M University, College Station, TX 77843, USA; cjwu@tamu.edu (C.-S.W.); dvillar77@gmail.com (D.V.); hyeewoon@tamu.edu (H.W.H.); shaodong.guo@tamu.edu (S.G.); 4 Department of Marine Bioindustry, Hanseo University, Seosan 31962, Korea; 5 Department of Biomedical Engineering, Texas A&M University, College Station, TX 77843, USA; gaharwar@tamu.edu; 6 Department of Veterinary Physiology and Pharmacology, Texas A&M University, College Station, TX 77843, USA; ytian@cvm.tamu.edu; 7 Department of Pediatrics, University of California, San Diego, CA 92161, USA; wenxian.fu@gmail.com; 8 Department of Metabolism and Aging, The Scripps Research Institute, Jupiter, FL 33458, USA; rgsmith@scripps.edu

**Keywords:** growth hormone secretagogue receptor (GHS-R), glucose-stimulated insulin secretion (GSIS), MIP-Cre/ERT, pancreatic islets, β cells, insulin secretion, insulin sensitivity

## Abstract

Growth hormone secretagogue receptor (GHS-R) is widely known to regulate food intake and adiposity, but its role in glucose homeostasis is unclear. In this study, we investigated the expression of GHS-R in mouse pancreatic islets and its role in glycemic regulation. We used Ghsr-IRES-tauGFP mice, with Green Fluorescent Protein (GFP) as a surrogate for GHS-R, to demonstrate the GFP co-localization with insulin and glucagon expression in pancreatic islets, confirming GHS-R expression in β and α cells. We then generated β-cell-specific GHSR-deleted mice with MIP-Cre/ERT and validated that GHS-R suppression was restricted to the pancreatic islets. MIP-Cre/ERT;Ghsr^f/f^ mice showed normal energy homeostasis with similar body weight, body composition, and indirect calorimetry profile. Interestingly, MIP-Cre/ERT;Ghsr^f/f^ mice exhibited an impressive phenotype in glucose homeostasis. Compared to controls, MIP-Cre/ERT;Ghsr^f/f^ mice showed lower fasting blood glucose and insulin; reduced first-phase insulin secretion during a glucose tolerance test (GTT) and glucose-stimulated insulin secretion (GSIS) test in vivo. The isolated pancreatic islets of MIP-Cre/ERT;Ghsr^f/f^ mice also showed reduced insulin secretion during GSIS ex vivo. Further, MIP-Cre/ERT;Ghsr^f/f^ mice exhibited improved insulin sensitivity during insulin tolerance tests (ITT). Overall, our results confirmed GHS-R expression in pancreatic β and α cells; GHS-R cell-autonomously regulated GSIS and modulated systemic insulin sensitivity. In conclusion, β cell GHS-R was an important regulator of glucose homeostasis, and GHS-R antagonists may have therapeutic potential for Type 2 Diabetes.

## 1. Introduction

Growth hormone secretagogue receptor (GHS-R) is a seven-transmembrane G-protein-coupled receptor (GPCR) expressed in the pituitary, various brain regions, and some peripheral tissues [1,2,3,4,5,6]. GHS-R is the recognized receptor of the hormone ghrelin [7], mediating ghrelin’s multifaceted effects on food intake, growth hormone secretion [2], proliferation, migration, and survival of epithelial cells [8,9]. Reports of GHSR-null mice by us and others indicated that GHS-R regulates the growth hormone secretion in the pituitary, gluconeogenesis and glycogenolysis in the liver, thermogenesis in the adipose tissues, and systemic insulin sensitivity [2,10,11,12].

Since GHS-R is expressed in brain regions controlling glucose sensing and in peripheral tissues regulating glucose production, uptake, and regulation, it is difficult to decipher the cell-autonomous effect of GHS-R in pancreatic β-cells using GHSR-null mice. GHS-R’s expression in pancreatic cells is controversial; some studies suggested that GHS-R is expressed in α, β, and δ cells of the pancreatic islets [3,4,5,6], while other studies suggested that GHS-R is not expressed in the β cells [5,6]. To address this critical knowledge gap, we first confirmed the GHS-R expression in β cells, then generated and characterized a novel mouse model with GHSR-deleted exclusively in β cells. The MIP-Cre/ERT line with INS1 promoter is currently considered a reliable β cell Cre line, with no known ectopic expression [13,14]. We thus used the MIP-Cre/ERT line to generate β-cell-specific GHSR-deleted mice (MIP-Cre/ERT;Ghsr^f/f^) and studied the role of β cell GHS-R in glucose homeostasis in these mice.

In the pancreas, GHS-R was suggested to mediate various ghrelin-induced functions, including inhibition of insulin secretion [15,16], stimulation of glucagon [17], and somatostatin [6]. At the same time, some reports suggested that some of ghrelin’s effects in peripheral tissues were independent of GHS-R [8,18,19,20,21,22]. We previously reported differential glycemic phenotypes in ghrelin-null and GHSR-null mice. During the glucose tolerance test (GTT), ghrelin-null mice had lower glucose and increased insulin levels, whereas old GHSR-null mice (Ghsr^−/−^) had lower glucose and decreased insulin levels [11,23]. Under a negative energy balance condition of 50% calorie restriction, we reported that both ghrelin-null and GHSR-null mice showed reduced blood glucose and failed to maintain normal blood glucose [24]. However, under positive energy balance condition of leptin-deficient diabetic ob/ob mice, we showed that global ablation of ghrelin increased insulin and improved hyperglycemia of ob/ob mice [23]; alternatively, global ablation of GHS-R decreased insulin secretion and aggravated hyperglycemia of ob/ob mice [25]. We recently reported that another peptide obestatin, which was derived from the same ghrelin gene, regulated insulin secretion via GHS-R in β cells [26]. Overall, GHS-R signaling was complex, and different peptides may utilize different signaling cascades. The direct effect of GHS-R in β cells was not clear; to better understand the effect of GHS-R on insulin secretion and glycemic regulation, a better model was needed.

Our study aimed to confirm the expression of GHS-R in pancreatic islets and determine the role of β cell GHS-R in glucose homeostasis. Using the GHS-R reporter line (Ghsr-IRES-tauGFP Knock-In), we demonstrated that the GFP protein, a surrogate of GHS-R, was co-expressed, with α and β cells in pancreatic islets. Using β-cell-specific GHSR-deleted mice (MIP-Cre/ERT;Ghsr^f/f^), we unequivocally demonstrated that GHS-R deficiency in β cells attenuated insulin secretion and improved insulin sensitivity.

## 2. Results

### 2.1. Validation of GHS-R Localization in Pancreatic Islets and Generation of MIP-Cre/ERT;Ghsr^f/f^ Mice

**Figure 1:**
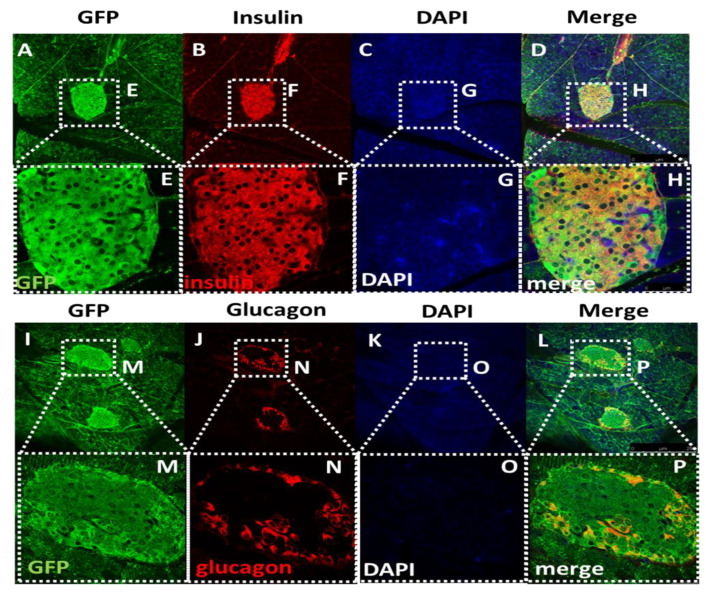
**Islet immunofluorescence staining of GFP, insulin, and glucagon.** (**A**–**D**) Images of islets within pancreatic tissue display the expression of (**A**) GFP, (**B**) insulin, (**C**) DAPI, and (**D**) the merged image in Ghsr-IRES-tauGFP Knock-In mice (magnification: 100×). The co-localization of GFP with insulin supported the growth hormone secretagogue receptor (GHS-R) expressed in β-cells. (**E**–**H**) Enlarged images of islet expression of GFP, Insulin, DAPI, and the merged image of the panel (**A**–**D**) (magnification: 400×). (**I**–**L**) Images of islet within pancreatic tissue display the expression of (**I**) GFP, (**J**) glucagon, (**K**) DAPI, and (**L**) the merged image. The co-localization of GFP with glucagon supports that GHS-R is expressed in α-cells. (**M**–**P**) Enlarged image of islet expression of GFP, glucagon, DAPI, and the merged image of the panel (**I**–**L**).

Since there was no reliable mouse antibody for GHS-R, we utilized GFP-GHSR reporter (Ghsr-IRES-tauGFP Knock-In) mice, where GFP was inserted into the GHS-R locus to serve as a surrogate of GHS-R. To determine whether GHS-R was expressed in pancreatic β cells, we investigated the co-localization of GFP with either insulin or glucagon expression in pancreatic islets. We detected high levels of GFP expression in the islets, clearly visualizing the co-localization of GFP with insulin- and glucagon-producing cells (Figure 1). As expected, we observed that insulin-expressing β cells were located inside the islets (Figure 1A–H), and glucagon-expressing α cells were located at the peripheries of the islets (Figure 1I–P). The co-expression of GFP with insulin- and glucagon-producing cells indicated that GHS-R was expressed in both β cells and α cells.

**Figure 2:**
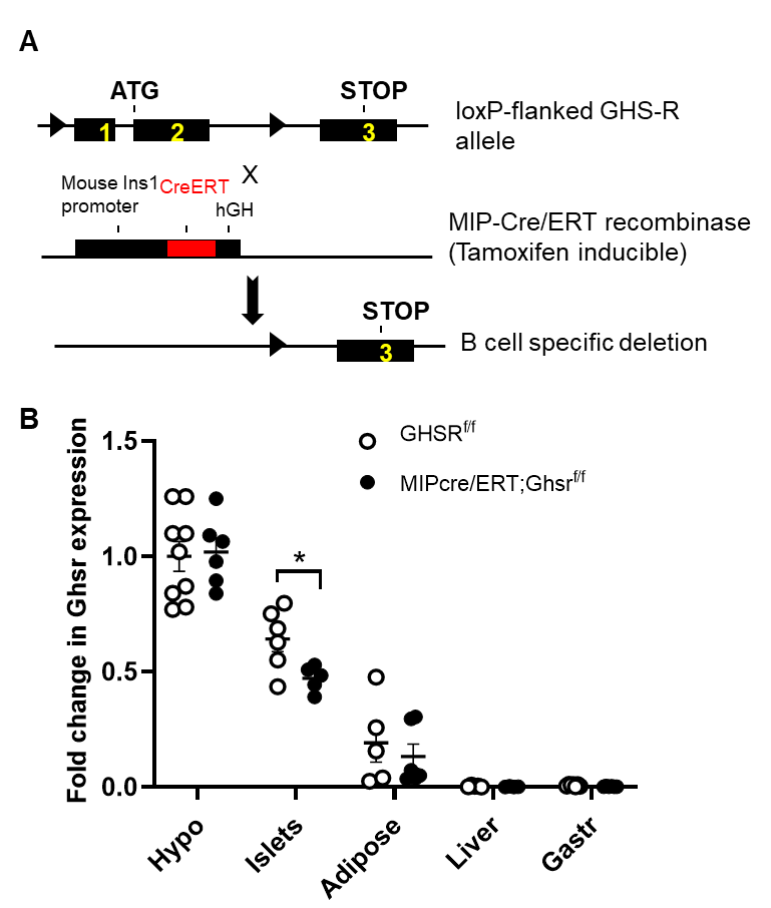
**Validation of β-cell-specific GHS-R deletion in MIP-CreERT;Ghsr^f/f^ mice.** (**A**) Schematic diagram of the recombination between the loxP-flanked GHS-R allele and MIP-CreERT. Tamoxifen induction of Cre ledsto recombination and excision of exons 1 and 2. The triangle represents the loxP sites. (**B**) Expression of GHS-R in various tissues of Ghsr^f/f^ and MIP-CreERT;Ghsr^f/f^ mice. * p < 0.05 Ghsr^f/f^ vs. MIP-Cre/ERT;Ghsr^f/f^. *n* = 3–9. Hypo: hypothalamus, Gas: gastrocnemius muscle, Fat—inguinal fat.

After establishing that GHS-R is expressed in β cells, we subsequently generated a β-cell-specific GHS-R knockout MIP-Cre/ERT;Ghsr^f/f^ mice by breeding our Ghsr^f/f^ mice [27] with the inducible MIP-Cre/ERT mice [14], as shown in Figure 2A. The MIP-Cre/ERT driven by the INS1 promoter was widely accepted as a good β-cell-specific Cre line, which was superior to most Cre lines that were previously used to target β cells [14]. To validate the mouse model, we measured the GHS-R expression in the brain and various peripheral tissues (at least 1 month after tamoxifen induction). As expected, the reduced GHS-R expression was only detected in the pancreatic islets of MIP-Cre/ERT;Ghsr^f/f^ mice, but not in other central and peripheral tissues tested (Figure 2B). The 30–34% reduction of GHS-R expression in the islets was likely primarily due to GHS-R deletion in the β cells, and the remaining GHS-R expression in the islets was likely from the GHS-R expression in other cells in the islets, such as α and/or δ cells.

### 2.2. β-Cell-Specific GHS-R Deletion Had No Effects on Energy Homeostasis

**Figure 3:**
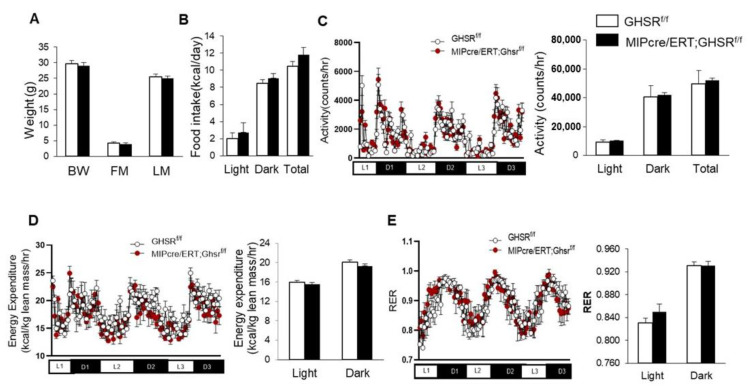
**GHS-R ablation in β cells had no impact on body weight and metabolic profile**. Five-to-five-point-five-month-old Ghsr^f/f^ and MIP-CreERT;Ghsr^f/f^ mice were subjected to the analysis of the comprehensive laboratory animal monitoring system. Metabolic parameters shown as column graphs were an average of 3-day recordings. (**A**) Body composition (BW: body weight, FM: fat mass, LM: lean mass); (**B**) food intake; (**C**) locomotor activity; (**D**) energy expenditure normalized by lean weight; (**E**) respiratory exchange ratio. *n* = 4.

We used Ghsr^f/f^ and MIP-Cre/ERT;Ghsr^f/f^ mice between 5 and 5.5 months old to evaluate the effect of β-cell-specific GHS-R deletion on energy homeostasis. Body weight and body composition were very similar between the Ghsr^f/f^ (control) and MIP-Cre/ERT;Ghsr^f/f^ mice (Figure 3A). To assess the metabolic effects of GHS-R deficiency in β cells, we performed an indirect calorimetry analysis. We observed that food intake, physical activity, and energy expenditure of the MIP-Cre/ERT;Ghsr^f/f^ mice were similar to the control mice (Figure 3B–D). The mice also had similar preferences for energy sources as depicted by the respiratory exchange rate (RER; Figure 3E). Taken together, this data suggested that β-cell-specific GHS-R ablations did not affect energy homeostasis, as evidenced by no effects on body weight, body composition, and metabolic profile.

### 2.3. β-Cell-Specific GHS-R Deletion Regulated Glucose Homeostasis

**Figure 4:**
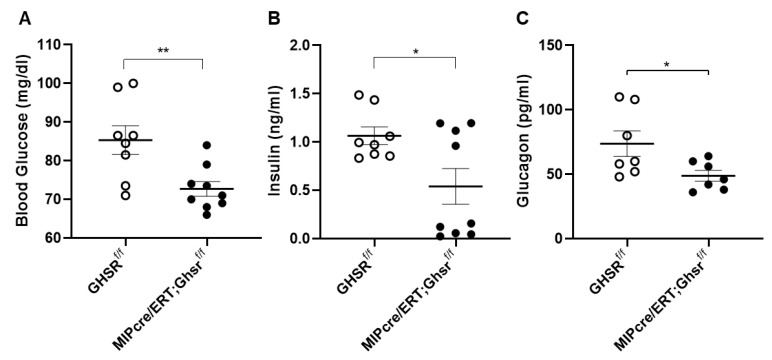
**GHS-R ablation in β cells reduced fasting glucose, insulin, glucagon.** Four-month-old mice were fasted overnight to measure (**A**) glucose and (**B)** insulin. (**C**) Five-month-old mice were fasted overnight, and serum glucagon was measured. *n* = 8–9. * *p* < 0.05, ** *p* < 0.01, Ghsr^f/f^ vs. MIP-CreER;Ghsr^f/f^.

To determine the role of GHS-R on the glycemic regulation in β cells, we assessed glucose and insulin levels, glucoregulatory hormones, glucose tolerance, and insulin sensitivity. Similar to our global GHSR-ablated mouse model [11], the β-cell-specific GHSR-deleted mice demonstrated reduced glucose and plasma insulin after overnight fasting (Figure 4A,B). The MIP-Cre/ERT;Ghsr^f/f^ mice also showed lower-fasting blood glucagon levels compared to the control mice (Figure 4C).

**Figure 5:**
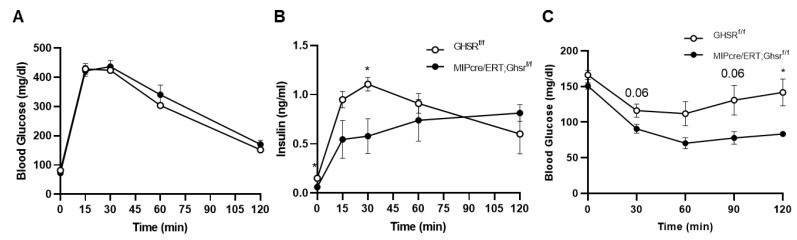
**GHS-R ablation in β cells altered insulin secretion and sensitivity**. (**A**,**B**) Three-point-five-month-old Ghsr^f/f^ and MIP-CreER;Ghsr^f/f^ mice were fasted overnight, and a glucose tolerance test was performed. Glucose and insulin were measured. *n* = 5. (**C**) Seven-month-old mice were fasted for 6 h and an insulin tolerance test was performed. *n* = 4. * *p* < 0.05 Ghsr^f/f^ vs. MIP-Cre/ERT;Ghsr^f/f^.

To test whether β cell GHS-R regulates insulin secretion under glucose load, we performed a glucose tolerance test on Ghsr^f/f^ and MIP-Cre/ERT;Ghsr^f/f^ mice. Although the changes in blood glucose after glucose stimulation were similar between the genotypes (Figure 5A), the insulin levels were significantly lower in MIP-Cre/ERT;Ghsr^f/f^ mice, most pronounced at 30 min (Figure 5B). To test whether β cell GHS-R regulates insulin sensitivity, we fasted Ghsr^f/f^ and MIP-Cre/ERT;Ghsr^f/f^ mice for 6 h and then performed the insulin tolerance test. MIP-Cre/ERT;Ghsr^f/f^ mice showed overall lower blood glucose compared with Ghsr^f/f^ controls, significant at 1 h post-insulin administration (Figure 5C), supporting that MIP-Cre/ERT;Ghsr^f/f^ mice had improved insulin sensitivity. Reduced insulin levels in GTT (Figure 5B) suggested that less insulin was required to maintain blood glucose in MIP-Cre/ERT;Ghsr^f/f^ mice, further supporting enhanced insulin sensitivity.

### 2.4. GHS-R in β Cells Attenuated Glucose-Stimulated Insulin Secretion In Vivo and Ex Vivo

**Figure 6:**
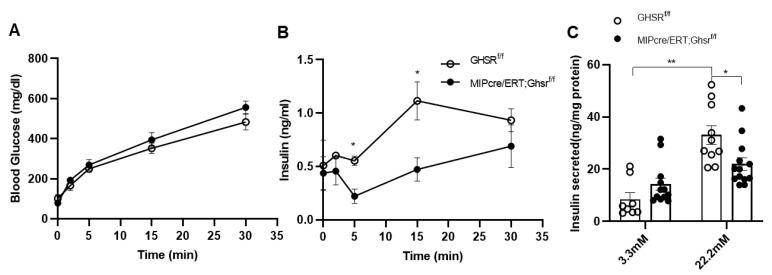
**GHS-R ablation in β cells reduced glucose-stimulated insulin secretion.** (**A**,**B**) Eight-month-old mice were fasted overnight, and in vivo glucose-stimulated insulin secretion (GSIS) was performed. Glucose and insulin were measured for, first, 30 min. *n* = 4–5. (**C**) Islets of 6-month-old Ghsr^f/f^ and MIP-CreER;Ghsr^f/f^ mice were treated with 3.3 mM or 22.2 mM glucose for 2 h. Insulin secretion was measured after 2 hours. The insulin levels were normalized to the total protein content of islets, *n* = 4–6. * *p* < 0.05, Ghsr^f/f^ vs. MIP-CreER;Ghsr^f/f^; ** *p* < 0.01, Ghsr^f/f^ at 3.3mM vs. 22.3 nM.

Normal β cell biology is characterized by biphasic insulin secretion patterns, namely the first phase and second phase of insulin secretion. Since a regular glucose tolerance test (2.0 g/kg glucose for 2 h) cannot easily distinguish between the biphasic insulin secretion patterns, we performed a 30 min 3.0 g/kg glucose-stimulated insulin secretion (GSIS) tests in vivo. Like our observation in the 2 h GTT, blood glucose was unchanged between the control and MIP-Cre/ERT;Ghsr^f/f^ mice (Figure 6A). Interestingly, the plasma insulin of MIP-Cre/ERT;Ghsr^f/f^ mice was significantly lower at 5′ and 15′ after glucose injection (Figure 6B). This suggested that β cell GHS-R had a major effect on first-phase insulin secretion.

To further assess if the effect of GHS-R on insulin secretion was a direct result of GHS-R deletion in β cells, we isolated pancreatic islets from the Ghsr^f/f^ and MIP-Cre/ERT;Ghsr^f/f^ mice and treated them with different concentrations of glucose (Figure 6C). Insulin secretion was measured after 2 h of glucose treatment. Under basal glucose (3.3 mM) conditions, insulin secretion was similar between the groups; under hyperglycemic conditions (22.2 mM), insulin secreted from MIP-Cre/ERT;Ghsr^f/f^ islets was significantly lower compared to that of control islets. Overall, these data supported that GHS-R in β cells regulated GSIS both in vivo and ex vivo.

## 3. Discussion

In this study, we demonstrated that GHS-R was expressed in α and β cells using a GHSR/GFP reporter mice (Ghsr-IRES-tauGFP Knock-In), and we investigated the β-cell-specific role of GHS-R in regulating insulin secretion using a novel mouse line MIP-Cre/ERT;Ghsr^f/f^. Ghsr-IRES-tauGFP Knock-In mice enabled us to assess the location of GHS-R in the pancreas using GFP signals as a surrogate for GHS-R. We visualized the co-localization of GFP with insulin- and glucagon-expressing cells in pancreatic islets, indicating that GHS-R was expressed in β and α cells (Figure 1). Our immunofluorescence results showed that insulin was primarily expressed in the center of the islets and glucagon was located in the periphery of the islets; these were consistent with the literature [28] and corroborated with previous reports in rats and humans that GHS-R was expressed in islet β cells [29,30]. Our finding of GHS-R and glucagon co-localization was also consistent with previous reports that GHS-R was expressed in mouse α cells [17]. Recently, single-cell transcriptome studies in mouse and human islets suggested GHS-R was expressed in δ cells [5,6,31]. During the time we conducted this study, we did not investigate the expression of somatostatin; hence, we cannot comment on the colonization of GHS-R and somatostatin in δ cells.

Many Cre mouse lines for the pancreas exhibited ectopic expressions in neuronal tissues [32]. The INS2 promoter line exhibited widespread ectopic expressions in the brain, and the Pdx1 promoter line showed ectopic expressions in the hypothalamus [14]. Impaired islet functions reported in the commonly used pancreas-specific Cre lines such as Pdx1-Cre^Late^, RIP-Cre, and MIP-GFP were attributed to the human growth hormone (hGH) minigene in the transgenic cassettes of mice [33]. The RIP-Cre mouse model had been shown to affect glucose tolerance in the mice even in the absence of genes targeted by loxP sites [34]. MIP-Cre/ERT line utilizes a fragment of the mouse insulin-1 gene to drive the Cre expression; it is one of the few limited β-cell-specific Cre lines available without ectopic expression [13,14]. However, the MIP-Cre/ERT transgenic line also had the hGH gene, and some recent studies demonstrated the hGH hormone expression in the MIP-Cre/ERT islets and brain [35,36]. Despite the hGH expression, it was reported that MIP-Cre/ERT mice on a chow diet had functionally normal β cells and exhibited similar glucose tolerance and insulin sensitivity [13,35]. Similar to tamoxifen-treated C57BL/6 mice, which did not alter glucose homeostasis during oral glucose tolerance test (OGTT) and GSIS in the isolated islets [37], tamoxifen induction of MIP-Cre/ERT mice also did not affect the glucose metabolism [13]. All previous reports on the MIP-Cre/ERT line suggested that the MIP-Cre/ERT transgenic mouse was a good mouse line for generating β-cell-specific recombination.

During the validation of our model, we detected that the GHS-R expression was only suppressed in the islets of MIP-Cre/ERT;Ghsr^f/f^ mice, not in the hypothalamus of the brain and other peripheral tissues tested (Figure 2). However, we only detected a 30–34% reduction of the GHS-R expression in the islets of MIP-Cre/ERT;Ghsr^f/f^ compared with Ghsr^f/f^ islets. The islets were primarily made of five types of endocrine cells: α, β, γ, δ, and ε cells. The reduced expression in the islets of MIP-Cre/ERT;Ghsr^f/f^ mice possibly reflected the GHS-R deletion only in β cells. The remaining GHS-R expression in the islets of MIP-Cre/ERT;Ghsr^f/f^ mice was likely contributed by the following factors: (1) GHS-R expression in other cell types such as α and δ cells; (2) low targeting efficiency of MIP-Cre/ERT line in β cells. The efficiency of the recombination of the MIP-Cre/ERT line was previously reported to be between 75–92%, and it appeared to depend on the dosage of the tamoxifen used for the induction [13,37]; (3) low level of expression of the INS1-Cre^ERT^ allele and the increased level of methylation compared with the wild-type allele contributing to inefficient deletion of the gene [38]. In prior work, we demonstrated that obestatin augments GSIS through GHS-R in β cells using various models, including GHS-R knockdown INS-1 cells (GHS-R antagonist, GHS-R siRNA), as well as GHSR-ablated pancreatic islets from global Ghsr^-/-^ mice [26]. The treatment of the MIP-Cre/ERT;Ghsr^f/f^ mouse islets with obestatin significantly attenuated the obestatin-induced GSIS in the control islets (Ghsr^f/f^ mice), but not MIP-Cre/ERT;Ghsr^f/f^ islets, supporting GHS-R had a critical role in β cells [26]. These results were consistent with our immunofluorescence staining data (Figure 1): together, these supported that both the expression and function of GHS-R were suppressed in β cells of the MIP-Cre/ERT;Ghsr^f/f^ mice.

Previously, we gained valuable information regarding various functions of GHS-R using the global Ghsr^-/-^ mice. We reported that young Ghsr^-/-^ mice had a normal body weight, body composition, and food intake, whereas the older Ghsr^-/-^ mice had a lower body weight and fat without a change in food intake [11,19]. Our data indicated that GHS-R played a role in the energy homeostasis during aging GHS-R deficiency augmented thermogenesis, which increased the energy expenditure, thus older Ghsr^-/-^ mice were leaner [11,19]. Using GHSR-null mice, we also demonstrated that GHS-R was important for the regulation of glucose sensing and insulin sensitivity. GHSR-null mice exhibited reduced glucose and insulin during overnight fasting and reduced glucose during 50% calorie restriction [24]. However, with the global Ghsr^-/-^ mouse model, we cannot reliably determine if the effects of GHS-R on glucose homeostasis were directly mediated by pancreatic β cells. MIP-Cre/ERT;Ghsr^f/f^ mice, where GHS-R was exclusively deleted in β cells, served as a more reliable tool to investigate the cell-autonomous effect of GHS-R in pancreatic β cells.

In this study, we assessed the whole-body glucose homeostasis of MIP-Cre/ERT;Ghsr^f/f^ mice and Ghsr^f/f^ controls after tamoxifen induction. To control for any possible effect of tamoxifen on glucose tolerance, we gavaged both Ghsr^f/f^ (control) and MIP-Cre/ERT;Ghsr^f/f^ mice with the same dose of tamoxifen for the same period. Like the global Ghsr^-/-^ mouse, MIP-Cre/ERT;Ghsr^f/f^ mice exhibited no change in body weight, body composition, food intake, and energy expenditure (Figure 3). Interestingly, MIP-Cre/ERT;Ghsr^f/f^ mice exhibited a reduced first-phase insulin secretion upon the glucose challenge during GTT and in vivo GSIS without much effect on blood glucose, suggesting an improved insulin sensitivity (Figure 5A,B and Figure 6A,B). The reduced first-phase insulin secretion had an important implication on diabetes; it generally represented exhaustion of β cells from a prolonged period of compensation for insulin resistance. However, since our insulin tolerance test suggested that the MIP-Cre/ERT;Ghsr^f/f^ mice improved insulin sensitivity (Figure 5C), it was possible that in this mouse model, less insulin was required to maintain glucose. To confirm whether the reduced GSIS observed was a direct effect of GHS-R in β cells, we further isolated the islets and performed GSIS ex vivo. Like our results in vivo, the islets from MIP-Cre/ERT;Ghsr^f/f^ mice secreted significantly less insulin compared to control wild-type (WT) islets under high glucose stimulation (Figure 6C). The MIP-Cre/ERT;Ghsr^f/f^ mice were able to maintain a normal glucose response, despite the reduced insulin secretion caused by GHS-R deficiency in β cells, which suggested that the glycemic control of MIP-Cre/ERT;Ghsr^f/f^ mice was effectively compensated by improved insulin sensitivity. Increased insulin sensitivity was associated with hypoinsulinemia in mouse models and humans [39,40]. This increased insulin sensitivity observed in MIP-Cre/ERT;Ghsr^f/f^ mice might be a compensatory response to reduced insulin secretion under hyperglycemic conditions. Reduced insulin secretion observed in MIP-Cre/ERT;Ghsr^f/f^ mice may suggest that there was a reduced need for insulin to maintain glucose homeostasis or may be due to some other factors such as (a) indirect effect of other insulinostatic hormones in the mice [41,42,43]; (b) impaired integrity of islet microvasculature leading to a reduced GSIS [44].

Insulin and glucagon work in concert to maintain glucose in a physiological range. In humans, fasting glucagon and insulin sensitivity have a nonlinear inverse relationship, such that fasting glucagon increases exponentially with decreasing insulin sensitivity [45]. Similarly, MIP-Cre/ERT;Ghsr^f/f^ mice were insulin sensitive and showed lower fasting glucagon (Figure 4C). It was recently reported that the islets of MIP-Cre/ERT mice expressed the hGH hormone, which led to the production of serotonin [35,36]. Serotonin could inhibit the glucagon secretion from α cells and increase the insulin secretion from β cells in a normal pancreas [46]. It was possible that serotonin in islets might be altered in the MIP-Cre/ERT mice, which was partly responsible for the reduced serum glucagon observed in MIP-Cre/ERT;Ghsr^f/f^ mice. It would be beneficial in future studies to elucidate the molecular mechanisms leading to improved insulin sensitivity in the mice and evaluate the hGH expression and serotonin production in islets of MIP-Cre/ERT;Ghsr^f/f^ mice.

With the current study, we cannot determine whether the phenotype observed was also contributed to by the reduced paracrine action of ghrelin in the islets and/or the loss of the constitutive activity of GHS-R in β cells. The paracrine role of ghrelin on insulin secretion was challenging for the following reasons: (a) the ghrelin-secreting cells were different in humans and rodents, which made it difficult to compare data; (b) the expression of the critical ghrelin acylation enzyme ghrelin O-acyltransferase (GOAT) was low in the pancreas, which limited active ghrelin in islets [47]. GHS-R1a had a very high constitutive activity and could signal at 50% of its maximum capacity even in the absence of its ligand [48]. Further studies of circulating ghrelin, constitutive activity of β cell GHS-R, and GHS-R signaling in other islet cell types such as δ cells [5,6] could be beneficial.

There were several confounding reports in the literature regarding the role of GHS-R in GSIS. Kurashina et al. reported that the treatment of GHSR-null mice that re-expressed GHS-R in β cells with a GHS-R antagonist led to increased insulin secretion [49]. This discrepancy in comparison to our data could be due to differences in the mouse models and/or the protocols used to study GSIS. The GHSR-null mice re-expressing GHS-R in β cells in Kurashina et al. utilized a rat insulin promoter-driven Cre recombinase (Ins-Cre), which is known to have an ectopic expression in the brain and muscles [49]. Further, in our study, islets were allowed to recover from the collagenase shock after the isolation procedure [50] by incubating them overnight in RPMI-1640, whereas Kurashina et al. used fresh islets [49]. Fresh islets were extremely susceptible to small changes in collagenase isolation procedure and may exhibit altered islet functions if not performed appropriately [51]. Lastly, we used 4.5–6.5-month-old mice, and they used 2–2.5-month-old mice.

Our study had some limitations that need to be addressed in the future. There might be a significant difference in the levels of GHS-R suppression in the individual MIP-Cre/ERT;Ghsr^f/f^ mouse, which resulted in a large variance in the glycemic phenotype observed (Figure 4). This could be due to insufficient tamoxifen dosage and/or treatment period, or variable deletion efficiency due to incomplete Cre recombination in the mice. To reduce the variance in GHS-R suppression between animals in each group, a non-inducible system could also be considered in the future. Previous studies of MIP-Cre/ERT mouse models reported no significant effect of the hGH expression on glucose tolerance and insulin sensitivity [5,33], so we only included Ghsr^f/f^ mice as controls. To be certain that the hGH cassette had no impact on the phenotype, tamoxifen-treated MIP-Cre/ERT mice would be a good additional control to include along with the Ghsr^f/f^ mice. Furthermore, since a growing body of evidence suggested that GHS-R was expressed in δ cells, and ghrelin-mediated insulin inhibition was mediated by somatostatin signaling [5,6], future studies should investigate the expression and signaling of GHS-R in δ cells as well.

In summary, our results showed that GHS-R was expressed in pancreatic β cells and had cell-autonomous effects on insulin secretion. GHS-R was an important regulator of glucose homeostasis, and β cell GHS-R had a critical role in insulin secretion and glycemic regulation. We believe that our results from MIP-Cre/ERT;Ghsr^f/f^ mice were a good representation of GHS-R deletion in β cells and were consistent with published data in global GHSR-null mice. β cell GHS-R likely had an important role in the pathogenesis of diabetes, and GHS-R antagonists may serve as an important therapeutic target for Type 2 Diabetes.

## 4. Materials and Methods

### 4.1. Animals

Ghsr^f/f^ mice were backcrossed to a congenic C57BL/6J background, as we recently reported [27], which were then used to breed with MIP-Cre/ERT mice [14] to generate β-cell-specific GHSR-deleted mice (MIP-Cre/ERT;Ghsr^f/f^). For the induction of GHS-R deletion, MIP-Cre/ERT;Ghsr^f/f^ and Ghsr^f/f^ control mice were gavaged with tamoxifen (4 mg/200 μL; T5648, Sigma-Aldrich, St. Louis, MO, USA) in peanut oil (P2144, Sigma-Aldrich, St. Louis, MO, USA) every alternate day for 5 days to minimize the toxic effect of tamoxifen. In Ghsr-IRES-tauGFP Knock-In mice, the GFP reporter was integrated into the GHS-R locus and translationally controlled by the same GHS-R promoter, so GFP was a good surrogate for GHS-R expression [52]. Animals were housed under controlled temperature and lighting (75 ± 1 °F; 12 h light-dark cycle from 6 a.m. to 6 p.m.) with free access to food and water. Mice were fed a regular diet (Cat no. 2920X, Harlan-Teklad, Indianapolis, IN, USA), and the diet composition was as follows: 16% of calories from fat, 60% from carbohydrates, and 24% from protein. All mice used in the experiments were age-matched congenic adult males that were housed and bred in a pathogen-free facility at the Baylor College of Medicine, and all methods were performed in accordance with the relevant guidelines and regulations. All experiments were approved by the Animal Care Research Committee of the Baylor College of Medicine, AN-2770 was approved on 7 August 2014.

### 4.2. Real-Time RT-PCR

Mouse pancreatic islets were isolated using an improved isolation protocol [53]. Total RNA from the hypothalamus and islets were isolated using Arcturus PicoPure RNA isolation kit (Applied Biosystems, Foster City, CA, USA) from fat, liver, and gastrocnemius muscles using TRIzol reagent (Invitrogen, Carlsbad, CA, USA) following the manufacturer’s instructions. cDNA was synthesized using the SuperScript III First-Strand Synthesis System (Invitrogen, Carlsbad, CA, USA). Real-time (RT)-PCR was performed on Bio-Rad Real-Time PCR Cycler (Bio-Rad Lab., Hercules, CA, USA) using SYBR Green PCR Master Mix according to the protocol provided by the manufacturer. Relative gene expression levels were normalized by 18S rRNA. We used a specific set of GHS-R 1α primers that flank the intron to distinguish the functional receptor GHS-R 1α from the truncated receptor GHS-R 1β [54]: forward primer 5′-GGACCAGAACCACAAACAGACA-3′, reverse primer 5′-CAGCAGAGGATGAAAGCAACA-3′.

### 4.3. Body Composition and Indirect Calorimetry Analysis

The whole-body composition (fat and lean mass) of the mice was measured by an Echo MRI-100 whole-body composition analyzer (Echo Medical Systems, Houston, TX, USA). Metabolic parameters were obtained using Oxymax (Columbus Instruments, Columbus, OH, USA) open-circuit indirect calorimetry system. To minimize the confounding effects of stress, mice were first individually caged in metabolic chambers and given free access to a regular diet/water for 3–4 days, then placed in metabolic cages for indirect calorimetry studies for an additional 5–6 days. The first 48 h were considered the acclimation phase, and the average data of the final 72 h were analyzed. Oxygen consumption (VO_2_; mL/kg/h), carbon dioxide production (VCO_2_; mL/kg/h), and locomotor activity (beam break counts) were measured. Respiratory exchange ratio (RER) and energy expenditure (EE, or heat generation) were calculated as previously described. Locomotor activity (on the *x*-axis) was measured using infrared beams to count the number of beam breaks during the recording period.

### 4.4. Glucose Tolerance Test (GTT), Glucose-Stimulated Insulin Secretion (GSIS), and Insulin Tolerance Test (ITT)

For GTT and GSIS, mice were fasted for 16 h (from 6 p.m. to 10 a.m.) prior to testing and then given an intraperitoneal (*i.p*.) injection of D-glucose (2.0 g/kg body weight for GTT, 3.0 g/kg body weight for GSIS). Blood glucose was measured by tail bleeds at different time points. For GTT, 25 µL blood from tails was collected in EDTA-coated tubes, and plasma samples were obtained by low-speed centrifugation for insulin measurement at 0, 15, 30, 60, and 120 min. For in vivo GSIS experiments, 25 µL blood from tails was collected in EDTA-coated tubes, and plasma samples were obtained by low-speed centrifugation for insulin measurement at 0, 2, 5, 15, and 30 min. The insulin tolerance test (ITT) was performed on mice fasted for 6 h (from 8 a.m. to 2 p.m.); humulin (Eli Lilly and Company, Indianapolis, IN, USA) was administered by *i.p.* injection (1 U/kg of Humulin) and blood glucose was measured at 0, 15, 30, 60, 90, and 120 min.

### 4.5. Blood Glucose, Insulin, Glucagon Measurement

Blood from the tails of mice was used to measure glucose using OneTouch Ultra blood glucose meter and test strips (LifeScan, New Brunswick, NJ, USA). Insulin was measured using Mouse Insulin ELISA 10*96 kit (Cat no. 10-1247-10, Mercodia, Uppsala, Sweden), and glucagon was measured using glucagon ELISA kit (Cat no. 10-1281-01, Mercodia, Uppsala, Sweden) according to the manufacturer’s instructions.

### 4.6. Immunofluorescence Staining of Pancreatic Islets

Six-month-old GFP-GHSR mouse was perfused with 10% formalin, tissues dissected and fixed overnight in 10% formalin at 4 °C, then stored in PBS + 0.1% sodium azide. The pancreas was sectioned into 100 μm with a vibratome. The following primary antibodies were used: Mouse anti-glucagon (Cat no. ab10988, 1:500, Abcam, Cambridge, UK), mouse anti-insulin (Cat no. MAB107, 1:500, Chemicon), and chicken anti-GFP (Cat no. NB100161402, 1:250, Novus Biologicals, Littleton, CO, USA). This was followed by secondary antibodies labeled with fluorochromes: Goat anti-Mouse-Alexa 594 (Cat no. A-11005, 1:500, Invitrogen, Carlsbad, CA, USA) and Goat anti-Chicken-Alexa 488 (Cat no. A-11039, 1:500, Invitrogen, Carlsbad, CA, USA). The immunofluorescence images of serial sections were captured on a Leica confocal microscope.

### 4.7. Ex Vivo Glucose-Stimulated Insulin Secretion (GSIS)

For insulin secretion assays, islets were isolated using Collagenase P (Cat no. 11249002001, Sigma-Aldrich, St. Louis, MO, USA) and incubated overnight [53]. The day before the assay, RPMI 1640 media containing 11.1 mM glucose were replaced with RPMI 1640 media containing 5.5 mM glucose and incubated overnight. On the day of assay, these media were further replaced by secretion media, HEPES Balanced Salt solution (HBSS; 114 mM NaCl, 4.7 mM KCl, 1.2 mM KH2PO_4_, 1.16 mM MgSO_4_, 20 mM HEPES, 2. 5 mM CaCl_2_, 25.5 mM NaHCO_3_, and 0.2% bovine serum albumin, pH 7.2) containing 3.3 mM glucose for 2 h. For GSIS assay, islets were incubated in 0.5 mL of the secretion media stated above, with a glucose concentration of 3.3 mM as a basal condition or 22.2 mM as a hyperglycemic condition. The conditioned cell media were collected for insulin assessment, and islets were harvested for protein normalization as we previously described [26].

### 4.8. Statistical Analysis

Graph-Pad Prism version 9.1.0 software (GraphPad Software, San Diego, CA, USA) was used. Two two-way ANOVAs with repeated measures or one-way ANOVA were used for statistical analysis. Data were represented as mean ± SEM, and statistical significance was set to a minimum of *p* < 0.05.

## Data Availability

Not applicable.
